# Enforcement of the Medical Law

**Published:** 1881-01

**Authors:** 


### Editorial.

### THE ENFORCEMENT OF THE MEDICAL LAW.

Sufficient time has not elapsed since the enactment of the medical law of 1880 to enable us to form anything like a correct judgment of its merits, nor of its influence in accomplishing the purpose for which it was passed. The central idea of the law, the registration of all duly authorized physicians, has been accepted by the profession, and generally complied with, especially in the large centres of population. In this city and county there have been registered two hundred and forty-five practitioners. Some interesting developments have also been made in regard to the *sources* of authority from which many practitioners derive their parchment. We do not desire to be hypercritical upon this point, losing sight of the professional merits and attainments of the individual in our haste to weigh in the balance his credentials. At the present, therefore, we are not concerned as much about those who have registered, however questionable may be the source from which some of the diplomas have been derived, as in regard to those who have failed to comply with this, the most important provision of the law.

Now all medical legislation, prior to the present law, has failed lamentably to accomplish the end designed to be attained in its enactment. The conviction in the minds of the most sagacious men in the profession has been well established, that medical science and its votaries, before they can expect to receive popular support and protection, must first deserve it. Whatever therefore may be the cause to which past failures may be attributed, it is very sure that the present proud position of the medical profession has been reached through its own inherent merits, and not through legislative enactments.

The provision of the new law, requiring all who practice physic and surgery to register their names in the office of the clerk of the county in which they reside, is salutary. The question is an interesting one, what measures should be adopted to reach those who fail to register. It would be idle to depend upon individual agencies and efforts to enforce the law. Too often such efforts would be spasmodic, or actuated by personal jealousy and rivalry. The law is in its intent too broad and comprehensive to be enforced with such motives.

The profession, in their corporate societies, should guide and direct the enforcement of the law. What better agency can be devised than the county medical societies?

We present this question at this time, inasmuch as throughout this State the annual meetings of the county medical societies will be held early in the present month, and the attention of the profession may then be directed to the various provisions of the law. If the matter is taken hold of by strong and sagacious men, the law can be made a valuable measure in protecting the profession and the public. To this end it is evidently the duty of those who possess ripe judgment and large experience to lend their counsel and advice in a matter which concerns the vital interests of the medical profession. We hope for successful results from the discussion of this subject, and also the adoption of wise and judicious measures.

